# Micro-Vesicles of *Moringa oleifera* Seeds in Heterozygous Rats for DAT Gene: Effects of Oral Intake on Behavioral Profile and Hematological Parameters

**DOI:** 10.3390/ijerph18052322

**Published:** 2021-02-26

**Authors:** Clelia Buccheri, Fabiana Festucci, Marina Potestà, Valentina Roglia, Roberta Bernardini, Antonella Minutolo, Carla Montesano, Walter Adriani

**Affiliations:** 1 Center for Behavioral Sciences and Mental Health, Italian National Institute of Health, 00161 Rome, Italy; clelia.buccheri@libero.it (C.B.); fabiana.festucci@student.univaq.it (F.F.); 2 Department of Biological Sciences, Tor Vergata University, 00133 Rome, Italy; marina.pote@gmail.com (M.P.); valentinaroglia@gmail.com (V.R.); antonellaminutolo@gmail.com (A.M.); montesan@uniroma2.it (C.M.); 3 Department of Biotechnological and Applied Clinical Sciences, University of L’Aquila, 67010 L’Aquila, Italy; 4 Interdepartmental Centre of Comparative Medicine, Alternative Techniques and Aquaculture, Tor Vergata University, 00133 Rome, Italy; roberta.bernardini@uniroma2.it

**Keywords:** *Moringa oleifera*, miRNA, cross-kingdom, dopamine transporter (DAT), behavioral profile, Forced Swimming test, spontaneous locomotor activity

## Abstract

Previous studies have shown multiple biological properties of *Moringa oleifera*, a plant native to Africa and Asia. In the present study, potential physiological properties of microvesicles extracted from *Moringa oleifera* seeds were assessed. For this purpose, we investigated behavioral profile and hematological parameters in a recent rat model characterized by dysregulation in dopamine transporter, a key regulator of dopaminergic system. Experimental design consisted of male Wistar-DAT rats aged between two and four months: wild-type (WT) (*n* = 5) and heterozygous (DATHET) (*n* = 4) control groups, which drank tap water; WT (*n* = 5) and DATHET (*n* = 6) groups which drank a solution of *Moringa* microvesicles and water (2: 68 mL per day), which was orally administered for two months. Rats were monitored for spontaneous locomotor activity on a 24/7 basis. In the early lit hours, treated DATHET subjects showed higher locomotor activity, proposing a sleep-delay effect of *Moringa*. In forced swimming test, WT subjects who took *Moringa* exhibited more depressive behavior. In DATHET rats, *Moringa* seemed to potentiate the struggle to find a way out, counteracting an initial panic. Hemoglobin and hematocrit underwent opposite changes in either genotype, supporting the opposite effects on behavioral phenotype observed. Future work is clearly needed to further explore these preliminary profiles.

## 1. Introduction

Cells can communicate with other neighboring cells by releasing extracellular vesicles (EVs). One type of extracellular vesicles consists of microvesicles (MVs) [1], which have the ability to transfer bioactive molecules, including proteins, DNA, mRNA, and miRNA in proximal and distal recipient cells [2,3]. Plant microvesicles (MVs) are a heterogeneous class of vesicles that contribute to plant growth and development, defense responses, and plant-microbe symbiosis [4]. Several studies showed that natural MVs, isolated from plants or milk, are readily taken up by intestinal cells in vitro and in vivo. [5,6]. 

These MVs may mediate plant-animal communication: they are involved in the transfer of miRNAs, protecting them from degradation, thus playing a relevant protective role against hostile environments following the transport into target cells [7]. MicroRNAs (miRNAs), a class of small single-stranded non-coding RNA with a length of 19–25 nucleotides, are thereby inhibiting the genetic expression at a post-transcriptional level. Recent experimental studies have reported that exogenous miRNAs of plants introduced with a vegetal diet are able to control target genes in organisms belonging to the animal kingdom [8].

*Moringa oleifera* is a plant belonging to the monogenus Moringaceae family. It is native to dry tropical forest in north-east India and foothills of the Himalayas, but it is also widespread in South Asia, Saudi Arabia, and in many regions of Africa [9].

Several components of the plant are edible (seeds, pods, flowers, and leaves) and have a high nutritional value; moreover, different parts of this plant (root, bark, pod, leaves, flowers, and seeds) are used in the treatment of several pathologies in African and Asiatic medicine. In these countries, *Moringa oleifera* is very important for its medicinal value: the extracts from *Moringa oleifera* exhibit multiple nutraceutical or pharmacological functions including anti-inflammatory, antioxidant, anti-cancer, hepatoprotective, neuroprotective, hypoglycemic, and blood lipid-reducing functions. Its beneficial functions are strongly associated with its phytochemicals such as flavonoids or isothiocyanates with bioactivity [10].

Potestà and colleagues reported that *Moringa oleifera* seed aqueous extract was able to regulate proliferation and apoptosis in cancer cells and this ability was associated with the presence of miRNAs [11]. In a later study, the authors characterized the bioactive components of MVs extracted from an aqueous extract of *Moringa:* and they demonstrated that MVs are not only able to carry miRNAs, but are also able to enter human cells and modulate activities related to viability and apoptosis in tumour cell lines [12].

Furthermore, *Moringa oleifera* seems to modulate the function of monoaminergic transmitters such as dopamine [13]. The catecholamine neurotransmitter dopamine controls many neurophysiological functions, such as: locomotion; the regulation of sleep-wakefulness; mood, by generating an overall psycho-physical wellness; reward process and motivation, acting a key role in psycho-biology of several types of addictions [14]. Since it carries out numerous functions, clearly its deregulation can cause the onset of different pathologies, such as Parkinson’s disease, schizophrenia, affective disorders, depression, bipolar disorder, and Tourette’s syndrome [15].

A key regulator of dopamine pathway is the dopamine transporter (DAT), which is a transmembrane transport protein present in the presynaptic axon terminal: its main physiological role is to regulate the concentration of dopamine in the synaptic cleft by operating its reuptake into presynaptic terminals. Recently, a novel DAT^trunk^ rat model was generated by molecular technology based on the action of zinc finger nucleases. DAT^trunk^ rats present both blunt alleles, meaning that they are in a homozygous state for the loss of functionality of the gene [16]. Compared to wild-type (WT) and heterozygous (DAT-HET) subjects, this rat model presented some peculiar phenotypic and behavioral characteristics: high spontaneous locomotor activity; deficits in working memory; clearly compulsive behavioral traits; dwarfism and weight reduced by 60%; a tendency to premature death and a tendency for females to give birth to few pups, which survive 24 h after birth [17,18].

Our colony of DAT^trunk^ rats represents a translational model useful for the study of ADHD (attention-deficit and hyperactivity disorder), as well as other human disorders that entail alterations in dopamine pathway [19,20,21]. Recently, we began to characterize heterozygous (HET) subjects instead of fully DAT^trunk^ ones, as a model for genetic vulnerability in this field [20,21].

In the current study, we chose to investigate the selective contribution of MVs extracted from *Moringa oleifera* seeds (aqueous extract) on behavioral aspects and on hematological parameters. To this purpose, WT and DATHET rats were left to drink MVs in tap water for two months.

## 2. Materials and Methods

### 2.1. In Vivo Experiment

#### 2.1.1. Ethical Note

All experimental procedures were approved by the Animal welfare survey board (OBA ISS) on behalf of the Italian Minister of Health (formal license 937/2018-PR and subsequent continuation 1008/2020-PR issued to WA). Procedures were carried out in close agreement with the directive of the European Communities Council (2010/63/EEC) and with the Italian law guidelines. We minimized animals’ suffering and we used as few animals as possible according to the regulations represented by the 3Rs principle.

#### 2.1.2. Experimental Groups

The colony has been maintained according to a classical breeding model (heterozygous x heterozygous parents); these animals were interbred for more than ten generations at Italian Institute of Technology (IIT, Genoa, Italy). Some progenitors were then sent to Italian National Institute of Health (Istituto Superiore di Sanità–ISS, Rome, Italy); here DAT^trunk^ male rats were interbred with Wistar-Han WT females (Charles River, Italy), obtaining a zero generation (G0) of new heterozygous progenitors. Parents, which were used to procreate present subjects as offspring, were G5 in our ISS colony. The experimental group consisted of 20 male rats born from our ISS colony, aged between 2 and 4 months and with a weight of 300–400 g measured at the beginning of experiments.

For experimental purposes, single rats were housed in cages equipped with control of enrichments, and placed in a housing room with environmental temperature (21 ± 1 °C) and relative humidity (60 ± 10%) and with an inverted light–dark cycle (lights went down at 6:30 a.m.). Throughout entire experiment, which lasted 2 months, each animal had standard food in pellet *ad libitum*. The experimental design was composed of four groups:

(1) Wild-type (WT) control group (*n* = 5), which had daily access to 70 mL of water;
(2) WT group treated with microvesicles (*n* = 5), which had daily access to 70 mL of a solution made by water and microvesicles of *Moringa*;
(3) Heterozygous (DATHET) control group (*n* = 4), which had daily access to 70 mL of water;
(4) DATHET group treated with microvesicles (*n* = 6), which had daily access to 70 mL of a solution made by water and microvesicles of *Moringa*.

Rats belonging to the wild-type group were generated and bred from both WT parents; rats belonging to the DATHET group were generated and bred from mating between DAT^trunk^ fathers and variable dams (i.e., partly wild-type and partly heterozygous mothers, in this way being either just heterozygous or also offspring of heterozygous). Recent unpublished data from our colony reveal that both these two conditions subserve the altered phenotype. We underline the additional role played by epigenetic components represented by variable maternal genotype, which is reflected in different cares given to the pups [22]. This is the usual breeding scheme in our ISS colony [20].

#### 2.1.3. Blood Sampling and Treatment

In order to evaluate hematological parameters, both before the treatment and at the end of treatment, each rat underwent an intracardiac blood draw. 

For each rat, an aliquot of about 1 mL of blood volume was used for obtaining serum and an aliquot of about 0.5 mL of blood volume was used to make the blood cell count.

### 2.2. Preparation and Administration of Microvesicles

#### 2.2.1. Preparation of Aqueous Seed Extract and Purification of Microvesicles

***Moringa oleifera* aqueous seed extract:***Moringa oleifera* mature seeds were harvested in Dschang District, West Cameroon (Africa) by the Cooperative of Medical Plant Producers SOCOPOMO. *Moringa oleifera* seeds were sun-dried and stored until use. The aqueous extract from seed powder was prepared and the concentration was calculated as fresh plant weight equivalent (FW), as previously described [12].

**Plant microvesicles purification and characterization:** Microvesicles were separated from a known *Moringa oleifera* aqueous extract concentration. For purification and filtering, we employed the usual protocol. Isolated microvesicles (MVs) were quantified using a Megamix-Plus SSC standard microparticle kit for ILV detection (Biocytex, Marseille, France) according to a previous study [12].

Through flow cytometry analysis, it has been possible to estimate MVs concentration as number of events/µL: 150.000 events have been registered in the gate including MVs of 100–500 nm, derived from Moringa oleifera aqueous extract (See Supplementary Figure S1). CytExpert 2.2 software (Beckman Coulter, Brea, CA, USA) was used for MVs’ quantification from Moringa oleifera aqueous extract, in three independent measurements for each of the three different MVs’ purifications. Every day for each rat, an average of 182,637 ± 37,589 microvesicles (contained in 1 g of Moringa oleifera seeds as FW) was administered.

#### 2.2.2. Oral Treatment Protocol

The water control group consisted of 9 rats (5 WT and 4 DATHET). The group treated with *Moringa oleifera* MVs consisted of 11 rats (of which there were 5 WT rats and 6 DATHET rats). Each week to prepare the treated extract, about 90 g (at least 1 g for each treated rat per week) of blended seeds were immersed in a beaker containing 310 mL of double-distilled water. The aqueous extract was first centrifuged 3 times for 10 min at 2500× *g* and finally a cycle for 30 min at 3000× g. It was then filtered by using a filter with a pores size of 0.22 μm (Minisart^®^) and then centrifuged for 5 min at 13,000× *g*. Finally, aqueous extract containing microvesicles was stored at +4 °C until use.

The volume of aqueous extract, obtained after final filtering, ranged between 115 and 154 mL; thus, the concentration of *Moringa* in the extract obtained each week ranged between 0.58 and 0.758 mg/µL. A volume of 2000 μL (containing no less of 182,637 ± 37,589 microvesicles, as calculated) was used and added to 68 mL of water for obtaining a solution of 70 mL to be daily dosed to each treated rat. Bottles containing 70 mL of such solution for treated rats (or containing 70 mL of tap water for water control rats) were placed every day on cages at 10 a.m. This final solution had no bitter flavor and was entirely drunk by all rats. Twenty-four hours later, any residual fluid content was measured. In this way, it was possible to control carefully the fluid consumption within 24 h. 

### 2.3. Behavioral Tests

In order to evaluate behavioral effects caused by the treatment, the four experimental groups were subjected to forced swimming test after about 40 days from the beginning of the treatment.

#### 2.3.1. Forced Swimming Test

It is classically used for the evaluation of the depressive-like behavior in animal models of psychiatric disorders and to access the efficacy of antidepressant drugs.

For this test, each animal was carefully placed in a cylindrical container (25 cm of diameter × 65 cm high) filled up with slightly warm water (24 °C ± 1). The level of water was 30 cm deep, in such a way that the animal could not touch the bottom of the container with its hind paws nor with its tail. The test session consisted of two exposures: the first one lasting 10 min in the morning and the second one, for a shorter period of 5 min, in the afternoon of the same day. 

The test was recorded using a SONY camera. Observations of videos were performed by a well-trained observer, using a scoring software (“The Observer^®^” by Noldus, NL), which allowed to calculate frequency, duration, and latency (i.e., time after the beginning of the observation for the first execution of the behavior) of several behaviors. 

The following behaviors were observed:

- “Swimming” (active swimming);
- “Struggling/Climbing” (powerful attempts to go out from the water, trying to climb along the cylinder walls, by using its four paws);
- “Diving” (underwater immersion);
- “Floating” (lack of movements except little movements with paws needed to maintain just nose and eyes above the water surface, in order to just allow to breathe with nose).

Swimming, struggling/climbing, and diving are indicating a responsive attitude and express the escape-directed activity. Swimming can be considered as an exploratory behavior in a coordinated way towards the surrounding environment, even if aimed to escape. Climbing the walls of the container by moving the four paws in an uncoordinated way can be considered as a panic symptom. Finally, movements aimed at simple floating was evaluated as an index of resignation (i.e., typical aspect of a depression-like profile) [23].

#### 2.3.2. Spontaneous Locomotor Activity

Rats were individually housed in single cages and placed in a recording rack, able to detect each movement (up to 20 movements per second) by means of a passive infrared sensor located on top of each cage. Data were recorded from a computer with a specific software (“ActiviScope” by TechnoSmart, Guidonia, Italy) and scores were automatically divided in bins of 60 min. Thereby, locomotor activity was monitored hour by hour within 24 h per day, 7 days a week.

### 2.4. Body Weight

Each rat was weighed before the beginning of the experiment and regularly every beginning of the week during the whole treatment period. WT group (*n* = 10 subjects) showed an average body weight of 426 g, while DATHET group (*n* = 10 subjects) had an average body weight of 370 g at basal timepoint. Increased or decreased weight effects were analyzed as variations, by calculating the difference between final and initial body weight for each group.

### 2.5. Statistical Analysis

All behavioral data were analyzed by using analyses of variance with repeated measurement (RM-ANOVA). The analyses were carried out using StatView II software (Abacus Concepts, Berkeley, CA, USA). Data were expressed as average ± SEM. Significance level was set at *p* ≤ 0.05, whereas significant tendencies were taken into account at 0.05 ≤ *p* ≤ 0.10, NS = not significant. Multiple post-hoc comparisons were carried out through Tukey HSD test: for each comparison, the threshold was calculated (MSD = minimally significant difference) beyond which a difference between the average values of groups can be evaluated as significant, with *p* < 0.05. 

In behavioral tests, in order to compare the four experimental groups, single ANOVAs were performed for each observed behavior. The genotype is a two-level “between” factor: Wild-type (WT) vs. Heterozygous (DATHET); the treatment is a two-level “between” factor: tap water vs. solution with microvesicles of *Moringa oleifera*. Time, instead, represents a “within” factor: its levels are repeated measures within the same individual.

As regards the forced swimming test, ANOVA analysis presented a 2 × 2 × 2 design: genotype (WT vs. DATHET) by treatment (water vs. MVs); the third factor “time” consisted of two levels (first vs. second half session).

As regards the monitoring of spontaneous locomotor activity, evaluated over the entire period of treatment, ANOVA analysis presented a 2 × 2 × 24 × 6 design: genotype (WT vs. DATHET) by treatment (water vs. MVs); the third factor “time” consisted of 24 levels (averaged daily hourly bins). In this analysis, a further “within” factor was introduced, which is a 6-level “decades” factor: in order to study in more detail the efficacy of treatment, all 60 days of treatment were divided in six intervals of ten days each (named “decades”). The average daily profile was calculated separately each ten days, yielding to one “average” day for each decade. 

Concerning the body weight, ANOVA analysis presented a 2 × 2 design: the genotype was a two levels factor (WT vs. DATHET), the treatment was a two level factor (water vs. MVs); the dependent variable was the body weight (as change over basal timepoint).

Regarding the blood parameters, ANOVA analysis presented a 2 × 2 design: the genotype was a two-level factor (WT vs. DATHET), the treatment was a two-level factor (water vs. MVs); dependent variables were the different blood parameters, expressed as change over basal level.

## 3. Results

### 3.1. Behavioral Test

#### 3.1.1. Forced Swimming Test (FST)

**Swimming**

Considering *frequency* of “Swimming” (which indicates a return to active swimming following each episode of Struggling) during the first session of morning, ANOVA analysis shows a significant difference between genotypes regardless of interaction with treatment (F[1,16] = 5.2; *p* = 0.03): WT subjects restarted to swim a higher number of times (12.1 ± 1.43), than DATHET subjects (8.3 ± 0.85), which rather displayed a panicked reaction (i.e., with much climbing and no return to swimming). Post-hoc analysis of Genotype * Treatment interaction shows that the difference between the averages of treated vs. control subjects just failed to overcome the threshold (MSD) value.

**Figure 1:**
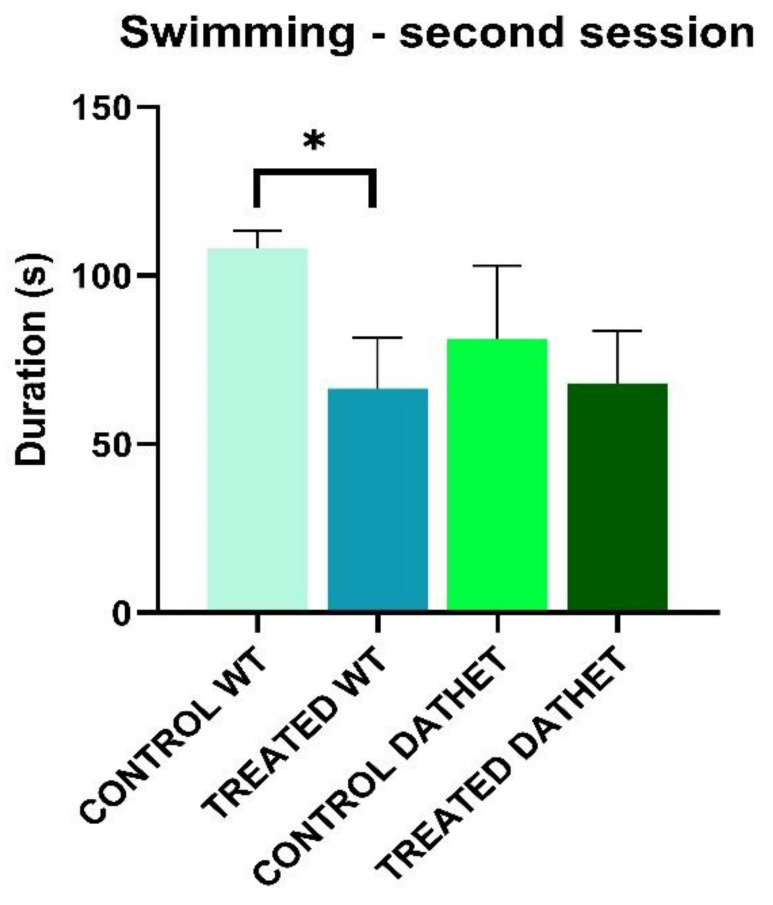
Forced swimming test was performed twice, in the morning and in the afternoon. The bar graph shows duration of swimming (0–150 s of afternoon session) for wild type (WT) and heterozygous (DATHET) control rats and rats treated with *Moringa*. Duration was shorter in WT treated subjects than water control subjects. Error bars represents standard errors of mean, SEM. Post-Hoc HSD Tukey: * *p* < 0.05 compared to control rats.

Considering *duration* of “Swimming” during the second session of afternoon, Time * Treatment interaction is significant (F[1,16] = 7.5; *p* = 0.01): the effects of treatment changed in relation to half session, regardless of genotype. In particular in the second half session, all treated groups spent less time swimming (67.3 ± 10.37) compared to water control groups (96.1 ± 10.40). Following post-hoc analysis for Time * Genotype * Treatment interaction, difference between treatments is significant when WT group is considered (MSD = 27.4; dF = 16, k = 4). In particular, compared to control WT group, a decrease of treated WT group has been observed, in the second half session (Figure 1).

**Struggling/Climbing**

**Figure 2:**
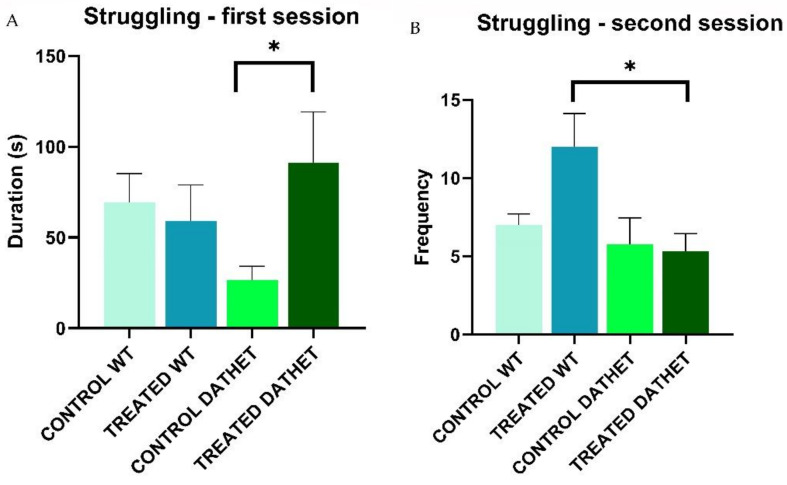
(**A**) Forced swimming test was performed twice, in the morning and in the afternoon. The panel shows duration of struggling (0–150 s of morning session) for WT and DATHET control rats and rats treated with *Moringa.* Duration was higher in DATHET-treated subjects than water control subjects. Error bars represents standard errors of mean, SEM. Post-Hoc HSD Tukey: * *p* < 0.05 compared to control rats. (**B**) The panel shows frequency of struggling (0–150 s of afternoon session) for WT and DATHET control rats and WT and DATHET rats treated with *Moringa*. Frequency was shorter in DATHET-treated subjects than WT-treated subjects. Error bars represents standard errors of mean, SEM. * *p* < 0.10 compared to control rats.

Considering *duration* of “Struggling” during the first session of morning, Time * Treatment interaction shows a significance in ANOVA analysis (F[1,16] = 5.6; *p* = 0.03). Through Tukey test, analysis, the Time * Genotype * Treatment interaction showed a better detail (MSD = 40.19; dF = 16, k = 3). When groups with different treatments were compared, DATHET-treated group with *Moringa* was significantly higher than water-control DATHET in the first half session of the forced swimming test (Figure 2 Panel A).

Considering *frequency* of “Struggling” during second session of afternoon, through ANOVA analysis, Genotype factor was significant (F[1,16] = 5.4; *p* = 0.03): regardless of treatment, WT group carried out transitions from swimming back to struggling with a higher frequency (7.5 ± 0.99) than DATHET one (4.2 ± 0.68), consistently with swimming frequency. Genotype * Treatment interaction seemed to show significant profiles through Tukey post-hoc analysis (MSD = 4.2; dF = 16, k = 2): when control DATHET and WT groups were compared, no difference was observed; if groups treated with *Moringa* were compared, the profile showed that DATHET genotype carried out struggling a lower number of times than WT genotype. Through ANOVA test, Time * Genotype * Treatment interaction tended to a significant value (F[1,16] = 2.9; *p*< 0.10); subsequent Tukey test confirmed this significance (MSD = 3.3; dF = 16, k = 4): when DATHET- and WT-treated groups were compared, WT subjects carried out struggling a higher number of times than DATHET ones in second session of forced swimming test (Figure 2 Panel B).

As a whole, *Moringa* generated opposite profiles: much more episodes of shorter duration in WT; longer episodes in DATHET. Panicked reaction typical of DATHET (i.e., with much climbing and fewer returns to swimming) was therefore potentiated.

**Floating**

Considering *duration* of “Floating” during the first session of morning, through ANOVA analysis, Genotype * Treatment interaction reveals a significance (F[1,16] = 4; *p* = 0.006): on the contrary, WT-treated group carried out floating with a higher duration (92 ± 27.03) than water controls of same WT genotype (42.2 ± 18.03); on the contrary, DATHET water control group carried out floating with a higher duration (97.7 ± 36.45) than the corresponding treated genotype (41.8 ± 11.27). 

**Figure 3:**
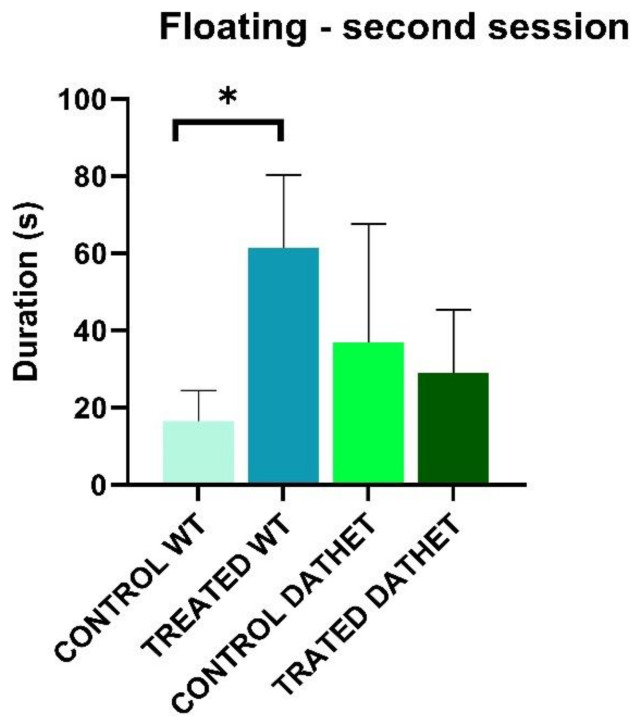
Forced swimming test was performed twice, in the morning and in the afternoon. The bar graph shows duration of floating (0–150 s of afternoon session) for WT and DATHET control rats and WT and DATHET rats treated with *Moringa*. Duration was higher in WT-treated subjects than water control subjects. Error bars represents standard errors of mean, SEM. Post-Hoc HSD Tukey: * *p* < 0.05 compared to control rats.

Considering *duration* of “Floating” during the second session of afternoon, Time * Treatment interaction was markedly significant (F[1,16] = 9.1; *p* = 0.008): regardless of genotype, control groups carried out floating similarly in the first and the second half session (21.2 ± 17.93); instead treatment with *Moringa* increased duration of floating in the second period of test (47.3 ± 11.98). Following post-hoc analysis for Time * Genotype * Treatment interaction (MSD = 28.05; dF = 16, k = 4), no effects came to light in DATHET group; instead, treatment was significant within WT groups in the second half session: treated rats carried out mainly floating, more than water control group (Figure 3). Depressive effect of *Moringa* took the form of floating in WT and of struggling in DATHET.

**Diving**

**Figure 4:**
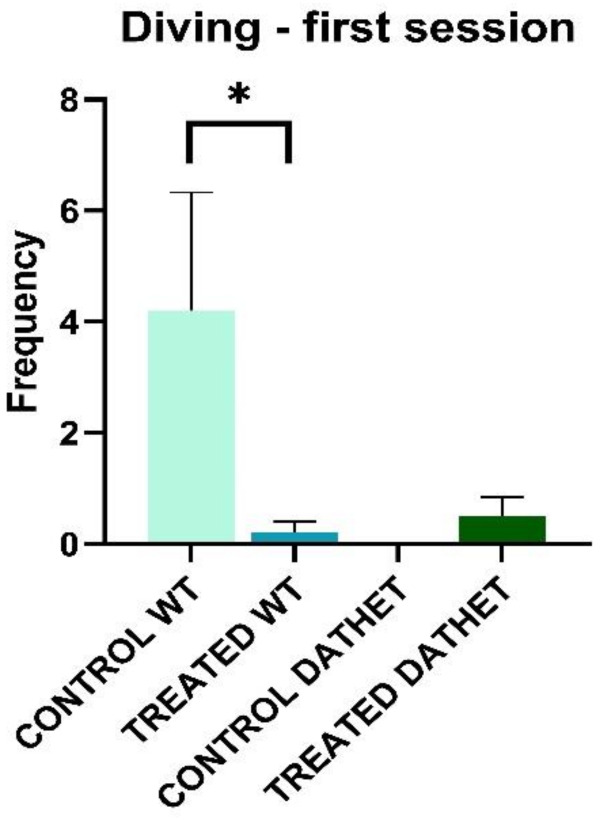
Forced swimming test was performed twice, in the morning and in the afternoon. The bar graph shows frequency of diving (0–150 s of morning session) for WT and DATHET control rats and rats treated with *Moringa*. Frequency was shorter in WT-treated subjects than water control subjects. Error bars represents standard errors of mean, SEM. * *p* < 0.05 compared to control rats.

Considering *frequency* of “Diving” during the first session of morning, Genotype * Treatment interaction was significant (F[1,16] = 4.1; *p* = 0.05), confirming the net prevalence of this behavior in WT control group than in all other three groups. Following Tukey test analysis (MSD = 1.63; dF = 16, k = 2), we confirmed the profile of Genotype * Treatment interaction (Figure 4).

Furthermore, Time * Genotype * Treatment interaction is significant but only in post-hoc approach. When groups are compared in relation to session, water control WT group carried out diving a higher number of times than WT-treated group and both DATHET groups, in particular in first session of forced swimming test (MSD = 2.26; dF = 16, k = 4).

#### 3.1.2. Spontaneous Locomotor Activity

**Figure 5:**
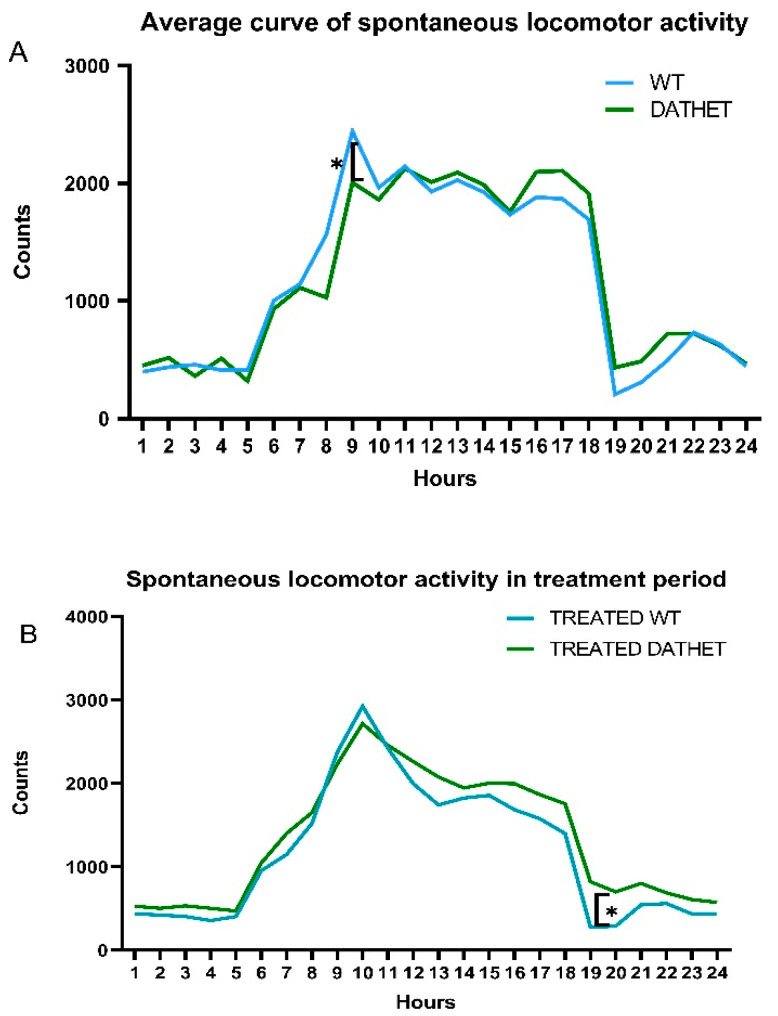
(**A**) Average 24-h curve of spontaneous locomotor activity of all experimental subjects during pre-treatment period (*n* = 20); (**B**) Average 24-h curve of spontaneous locomotor activity for *Moringa*-treated groups of either genotype (*n* = 5/6) during the whole treatment period.

Like many other mammalians, rats have an inverted circadian rhythm compared to that of most animals and humans [24]: the spontaneous locomotor activity in the baseline period shows a rapid increase starting from 6 a.m. (when the lights went off in the housing room and rats began to wake up) until a peak around 9 a.m.; a steady trend is then maintained; finally, a drastic decrease is observed around 7 p.m. (when lights went on and rats went to sleep) (Figure 5 Panel A).

From statistical analysis of spontaneous locomotor activity, in the basal pre-treatment period, Hours * Genotype interaction was not significant (F[23,414] = 1.3, *p* = 0.15). Moreover, through post-hoc test, there were no significant differences between WT and DATHET genotypes (MSD = 488.7; dF = 414, k = 7), except for 8:00–9:00 a.m. bins (2 h after lights went off, which represents the time of awakening): when WT subjects had a slightly higher locomotor activity.

Analyzing the treatment period of six decades, through the ANOVA test, we found a Decades * Hours * Genotype interaction (F[35,560] = 5.8; *p* = 0.01): during the collapsing of ten-days periods, the morning peak that was shown at 9 a.m. at basal and in water controls, in both treated groups was moving forwards. Thus, it can be proposed that treatment with *Moringa* may develop a similar delayed onset of circadian activities for WT and DATHET groups: this initial genotype difference at beginning of the phase of darkness got thinner and disappeared.

Finally, we have been looking at Hours * Genotype * Treatment interaction through post-hoc analysis (MSD = 434.7; dF = 112, k = 4): the treatment with MVs of *Moringa* showed a slight effect on treated subjects of DATHET genotype, increasing their activity at 7 a.m. and 8 p.m. (Figure 5 Panel B). In this case, a delayed onset of sleeping may be proposed.

### 3.2. Physiological and Hematological Parameters

At the end of treatment, both WT control and treated groups gained 76 g compared to initial weight; DATHET control and treated groups gained about 105 g. 

Hematological parameters of each experimental group were analyzed both at basal and final timepoints. By comparing values across a same group, as shown on Table 1, variations comprised between −10% and +10% (when compared to water controls) were observed. Significant variations of hemoglobin and hematocrit were part of the physiological range and they do not reveal any type of anemia or blood pathology [25].

A significant but not pathological difference was observed in percentage variation of number of *Red blood cells*, in relation to genotype, but regardless of treatment. Following analysis, ANOVA reveals that Genotype effect is significant (F[1,16] = 4.5; *p* = 0.05): in WT subjects there was a positive percentage variation, instead in DATHET subjects there was a negative percentage variation. Subsequent analysis with Tukey confirmed this Genotype * Treatment interaction: it was found that water control WT group showed a higher percentage variation (+13.2% ± 7.74) compared to water control DATHET group (−6.5% ± 7.93). This means that the number of erythrocytes increased at final time with reference to baseline value in WT subjects; on the contrary, in DATHET subjects, the number of erythrocytes decreased at final time with reference to baseline value. 

**Figure 6:**
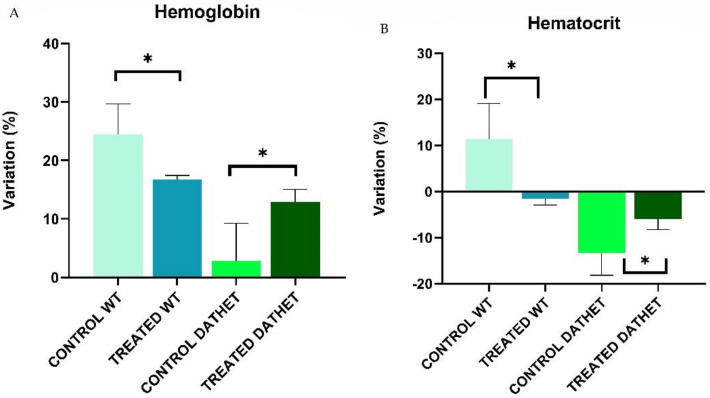
(**A**) Percentage variation of Hemoglobin (between end of treatment and baseline) in control rats and treated rats. The bar graph shows that percentage variation of hemoglobin was reduced in WT-treated subjects than water control subjects; DATHET-treated subjects showed a higher percentage variation compared to water control subjects. (**B**) Percentage variation of Hematocrit in control rats and treated rats. The bar graph shows that WT-treated subjects exhibited a more negative percentage variation than water control subjects; DATHET-treated subjects showed a reduced negative percentage variation compared to water control subjects. Error bars represents standard errors of mean, SEM. * *p* < 0.05 compared to control rats.

Regarding the quantity of *Hemoglobin* transported by red blood cells per volume of blood, following analysis by ANOVA, the Genotype effect was significant (F[1,16] = 10.7; *p* = 0.004): WT group showed a higher percentage variation (+20.6 % ± 2.79) compared to DATHET group (+8.8% ± 3.13). When comparing the two genotypes in relation to treatment, Genotype * Treatment interaction was significant (F[1,16] = 5.2; *p* = 0.03): WT-treated subjects (+16.7% ± 0.72) showed a slightly reduced percentage variation in hemoglobin compared to water control subjects (+24.4% ± 5.20); while DATHET-treated subjects (+12.9% ± 2.12) showed a higher percentage variation compared to water control subjects (+2.7% ± 6.46) (Figure 6 Panel A). 

Regarding *Hematocrit* (percentage volume occupied by red blood cells in relation to total blood volume) through analysis with ANOVA, the Genotype effect was clearly significant (F[1,16] = 9.8; *p* = 0.006): in WT group there was a positive percentage variation (+4.91% ± 4.29) compared to DATHET group, which showed a negative percentage variation (−8.88% ± 2.52). When comparing the two genotypes in relation to treatment, Genotype * Treatment interaction was significant (F[1,16] = 4.7; *p* = 0.04): in WT group treatment with MVs of *Moringa* led to a negative percentage variation (−1.48% ± 1.42) compared to control group, which showed a positive percentage variation (+11.32% ± 7.76); in DATHET group, instead, the treatment caused a reduced size of negative percentage variation (−5.93% ± 2.29) compared to water control group (−13.30% ± 4.86) (Figure 6 Panel B).

No significant percentage variation was found in other blood parameters.

## 4. Discussion

Previous studies with FST demonstrated that WT subjects try to escape by searching for a way out, by looking around and diving. While the present WTs acted through a smarter and more coordinated way, DATHET ones did so through an agitated and uncoordinated way. This made DATHET rats more similar to DAT^trunk^ ones [20]. The exposure to acute stress, represented by impossibility to escape and by unconditioned fear to drown, leads to the manifestation of a typical physiological reaction, classicaly termed “fight or run away” [26]. After a whole six weeks of exposure, treatment was confirmed to induce a depressant-like profile. In WT group, the treatment with *Moringa* MVs seems to induce a passive floating (i.e., a despair behavior), highlighted by an anomalous fragmentation or lack of reactivity. DATHET subjects were characterized by even more frantic escape-directed activity.

*Moringa* MVs, which were capable of acting on these emotional and behavioral aspects, seemed to possess a motivational relaxing effect, in WT rats, rather, for the already vulnerable DATHETs, it acted by than interacting with any initial panic phase. WT subjects who drunk *Moringa* showed a more passive and helpless behavior; treated DATHETs never spent a higher time in passive floating, but they showed a lower number of longer episodes of agitation, witnessing a more reactive form of depression.

Effects of MVs contained in *Moringa oleifera* seeds have been barely examined in regards to sleep. In our hands, a sleep-delay effect due to intake of *Moringa* MVs could be deduced. The DATHET subjects treated with *Moringa* MVs are characterized by hyper-locomotion during the first lit hours. This effect could be explained by a forward slipping of circadian rhythm: therefore, DATHET subjects may need more time before they go to sleep. In the first “dark” hours, both groups had delayed wake up, also. Alternatively, a different timing for *Moringa* intake could be suggested during the hours just preceding facility-lights turning on: sleep time may thus vary as a consequence of a differential drinking effect; in order to confirm this hypothesis, future experiments are needed to track when subjects drink during the day.

Treatment with MVs of *Moringa oleifera* did not involve any variation in body weight under conditions of normocaloric diet. The administration of *Moringa* MVs induced an opposite effect on the amount of hemoglobin and hematocrit. Since this effect did not cause the onset of anemia nor hyperproduction of red cells, we can exclude any pathological sequelae by long-term use of *Moringa* MVs. Our data suggest that a slight modulation of red cell production may well be one among the effects of *Moringa oleifera* intake both on humans and on rats.

## 5. Conclusions

Results described above may be due to the combination of genetic aspects (presence or not of one truncated DAT allele) and epigenetic impact (post-transcriptional genic silencing possibly caused by miRNA) thereon.

A similar study reported that the chronic oral treatment with ethanolic extract of *Moringa oleifera* leaves can alter the brain monoamines (norepinephrine, dopamine, and serotonin) in distinct brain areas in a rat model of Alzheimer’s disease [27]. The interest of our group was to investigate how microvesicles contained within seeds may exert any effect on the behavioral phenotype, as a function of a vulnerable dopaminergic pathway. 

According to our results, showing an opposite interaction between treatment and DAT genotype, we propose that such miRNA may act as key regulators of dopaminergic pathway. In heterozygous subjects the neurotransmitter is reuptaken in a less effective way within brain striatum, while the opposite is true for prefrontal cortex [20,21]. As a consequence, DATHET rats are more anxious and vulnerable to aversive states [21]. Prolonged drinking of *Moringa* MVs may induce a loss of multiple functionality and result in differential amounts of dopamine released into the synaptic space within distinct brain areas. Further work will address whether MVs-derived miRNAs actually cross the blood brain barrier.

In a future perspective, *Moringa oleifera* may be used as potential to support pharmacological therapy. For this purpose, further studies will be required for tracking the exact target sites of MVs’ miRNAs and to understand in detail the mechanism of action.

## Figures and Tables

**Table 1:** Main hematological parameters of WT- and DATHET-treated groups. For each blood parameter, range values, and final values are displayed. This table clearly shows the negligible variations (comprised between −10% and +10% when compared to water controls), preserving in a physiological range the values in both groups treated with MVs. The data are confirming the non-toxic effect of *Moringa*.

**White Blood Cells** Range Values = 3.0–8.0 10^3^/mm^3^		**Lymphocytes** Range Values = 1.2–26.4 10^3^/mm^3^		**Granulocytes** Range Values = 0.3–2.8 10^3^/mm^3^	
**WT**	**DATHET**	**WT**	**DATHET**	**WT**	**DATHET**
2.3 ± 0.33	1.7 ± 0.09	1.6 ± 0.23	1.2 ± 0.08	0.6 ± 0.09	0.5 ± 0.06
**Red Blood Cells** Range Values = 7.0–9.0 10^6^/mm^3^		**Hemoglobin** Range Values = 15.0–18.0 g/dL		**Hematocrit** Range Values = 35.0–55.0 %	
**WT**	**DATHET**	**WT**	**DATHET**	**WT**	**DATHET**
8.5 ± 0.04	8.8 ± 0.13	15.0 ± 0.15	15.5 ± 0.2	40.2 ± 0.45	41.1 ± 0.7

## Data Availability

Raw data are stored on a computer in the office of the corresponding author for five years, and can be made available upon request.

## Supplementary Materials

The following are available online at https://www.mdpi.com/1660-4601/18/5/2322/s1, Figure S1: **A**. Representative pseudo-dot plot (FSC-H vs. SSC-H) of standardized fluorescent (FITCA) nanosized particles of different sizes from the Megamix-Plus SSC kit used as a control for the analysis of the MVs. **B**. Representative pseudo-dot plot (FSC-H vs. SSC-H) of MVs extracted from MOES, used for the treatments [12].
