# Supplementary material for: Micro-Vesicles of Moringa oleifera Seeds in Heterozygous Rats for DAT Gene: Effects of Oral Intake on Behavioral Profile and Hematological Parameters
Source: Int J Environ Res Public Health. 2021 Feb 26;18(5):2322. doi: 10.3390/ijerph18052322 (PMC7956377; doi:10.3390/ijerph18052322)
Supplement: Supplementary file 1 [file ijerph-18-02322-s001.pdf]

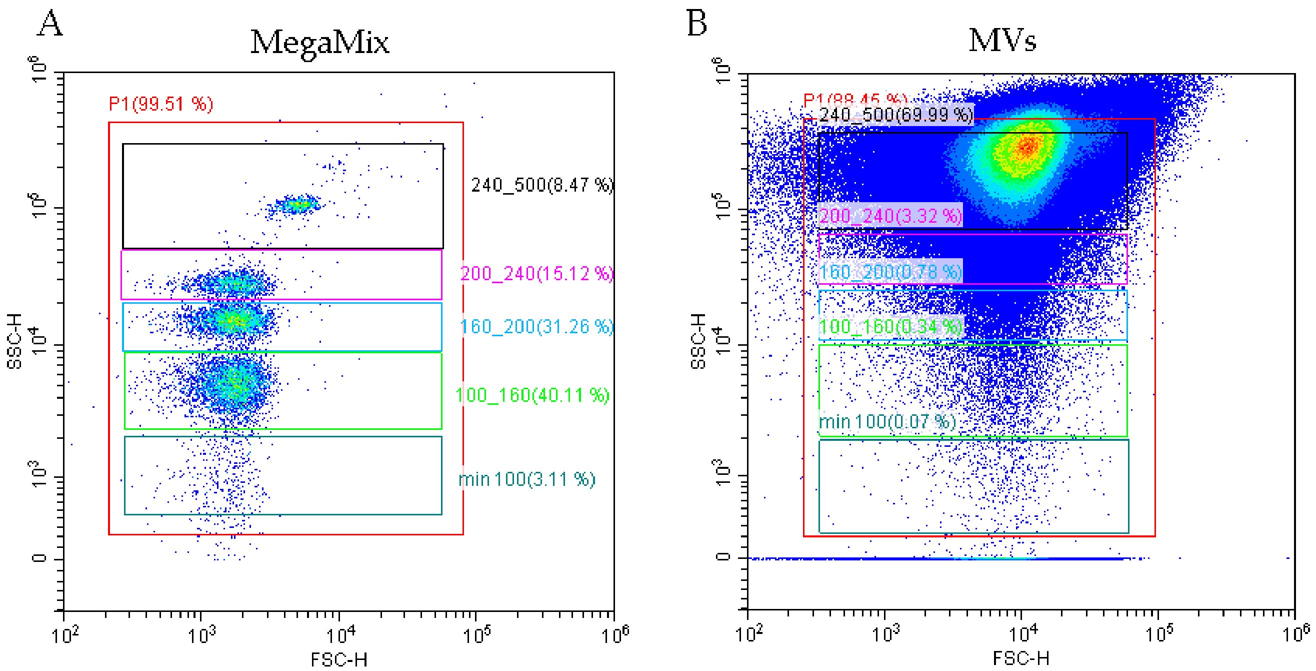

Supplementary figure. Representative pseudo-dot plot (FSC-H *vs* SSC-H) of standardized fluorescent (FITCA) nanosized particles of different sizes from the Megamix-Plus SSC kit used as a control for the analysis of the MVs. **B**. Representative pseudo-dot plot (FSC-H *vs* SSC-H) of MVs extracted from MOES, used for the treatments [12].
